# Albuminuria Is Associated with Left Ventricular Hypertrophy in Patients with Early Diabetic Kidney Disease

**DOI:** 10.1155/2014/351945

**Published:** 2014-08-20

**Authors:** Nan Wu, Weiwei Zhao, Kuanping Ye, Yintao Li, Min He, Bin Lu, Renming Hu

**Affiliations:** The Institute of Endocrinology and Diabetology, Huashan Hospital, Shanghai Medical College, Fudan University, 12 Middle Wulumuqi Road, Shanghai 200040, China

## Abstract

*Aims.* Left ventricular hypertrophy (LVH) and albuminuria are both markers for cardiovascular diseases (CVDs) in patients with type 2 diabetes mellitus (T2DM). We speculate that albuminuria in T2DM patients with early diabetic kidney disease (DKD) could predict LVH. *Methods.* 333 diabetic patients (219 non-DKD and 114 early DKD) were enrolled. The association between albuminuria and LVMI was examined using multivariate linear regression and logistic regression. *Results.* The rate of LVH was significantly higher in patients with early DKD versus those without DKD (57.0% versus 32.9%; *P* < 0.001). Multivariate linear regression analysis demonstrated that albuminuria status (no, micro-, and macroalbuminuria; *P* < 0.001), age (*P* < 0.001), systolic blood pressure (*P* = 0.0578), and the use of ACEI/ARB drug (*P* < 0.001) were independently associated with LVMI. The risks were substantially higher for LVH in the microalbuminuria group (odds ratio 2.473 (95% confidence interval 1.370–4.464)) and macroalbuminuria group (odds ratio 3.940 (95% confidence interval 1.553–9.993)) compared with that in non-DKD group. Concentric hypertrophy was the most common geometric pattern in patients with early DKD (36.0%), followed by eccentric hypertrophy (21.0%). *Conclusions.* Albuminuria is associated with higher LVMI and higher rate of LVH in patients with early phase DKD.

## 1. Introduction

Diabetic kidney disease (DKD) is a common microvascular complication of type 2 diabetes mellitus (T2DM) and may eventually require renal replacement therapy (RRT) in many patients. DKD is also an independent risk factor of cardiovascular diseases (CVDs) [1]. Left ventricular hypertrophy (LVH) is typically a response to increased afterload and is an independent risk factor for coronary heart disease, sudden cardiac death, and heart failure [2]. LVH is also associated with various metabolic abnormalities, such as central obesity, dyslipidemia, insulin resistance, and T2DM, even in the absence of hypertension [3, 4].

Albuminuria is an independent risk factor of rapid progression of chronic kidney diseases (CKDs) and in T2DM, a warning sign for DKD [5]. The level of urinary albumin excretion has been associated with CVDs in diabetic patients with CKD [6, 7].

Based on these findings, we speculate that the level of albuminuria is associated with LVH in T2DM patients in early stage of renal impairment, and we conducted a cross-sectional study to examine whether LVH is associated with LVH in T2DM patients with estimated glomerular filtration rates (eGFR) at >60 mL/min/1.73 m^2^.

## 2. Subjects and Methods

### 2.1. Participants

Adult T2DM patients with no or only mild impairment to the kidney were included in the study. Specifically, eGFR must be >60 mL/min/1.73 m^2^ and serum creatinine increase during the past 6 months must be <2-fold. Patients with any of the following conditions were not included: myocardial infarction within the past 6 months, a history of stroke or occlusive peripheral vascular disease, heart failure, uncontrolled thyroid diseases, active urinary tract infection, and any type of other renal diseases. Also, patients who had coronary artery bypass or angioplasty were not included. All subjects received periodic medical care as outpatients at the Huashan Hospital. Written informed consent was obtained from all participants. The study was approved by the institutional review board (IRB) of the Huashan Hospital.

### 2.2. Definition of Early DKD

DKD was defined as the presence of persistent albuminuria plus diabetic retinopathy in T2DM patients [8]. Persistent proteinuria was defined as urinary albumin-to-creatinine ratio (ACR) at >30 mg/g upon at least two consecutive visits. Early DKD was defined as DKD with eGFR at >60 mL/min/1.73 m^2^ [9]. eGFR was calculated using the 4-variable MDRD equation [10].

### 2.3. Echocardiography

Echocardiographic examination was performed with the patient in a partial left lateral decubitus position under two-dimensional guided M-mode using a Vingmed System 5 Doppler echocardiographic unit (GE Vingmed Ultrasound, Horten, Norway), as recommended by the American Society of Echocardiography [11]. Left ventricular mass (LVM, in unit of g) was calculated using the Devereux formula as follows: LVM = 1.04 × [(LVIDD (left ventricular internal diameter, diastolic) + PWTD (posterior wall thickness, diastolic) + IVSD (interventricular septum, diastolic))^3^ − LVIDD^3^] − 13.6 [12]. Relative wall thickness (RWT) was calculated as 2 × PWTD /LVIDD. Increased RWT was defined when RWT was >0.42 [13].

LVM index (LVMI) was derived by correcting LVM for body surface area. LVH was defined as follows: LVMI > 125 g/m^2^ for men and LVMI > 110 g/m^2^ for women [14]. LV geometry was considered “normal” if both RWT and LVMI are within the normal range, “concentric remodeling” with increased RWT but normal LVMI, “eccentric hypertrophy” with increased LVMI but normal RWT, and “concentric hypertrophy” with increased LVMI as well as increased RWT [13].

### 2.4. Others

Body mass index (BMI) was defined as weight in kilogram divided by square height in meter. Blood pressure (BP) was the average of two measurements in the supine position under a resting state using a mercury sphygmomanometer with an appropriate cuff on the left arm.

A 12-hour overnight fasting venous blood sample was collected for measurement of plasma glucose, insulin, HbA1c, creatinine, uric acid, total cholesterol, low-density lipoprotein cholesterol (LDL-C), high-density lipoprotein cholesterol (HDL-C), and triglycerides (TG) using standard protocol. Insulin resistance was estimated using homeostasis model assessment index-insulin resistance (HOMA-IR) [15]. First morning urine was collected once every month for three consecutive months.

### 2.5. Data Analysis

The primary interest in the data analysis was the relationship between LVMI and albuminuria level (no albuminuria and microalbuminuria versus macroalbuminuria) in T2DM patients. The analysis was carried out with a multivariate linear regression, using factors identified by the initial univariate linear regression. Microalbuminuria was defined as ACR between 30 and 300 mg/g; macroalbuminuria was defined as ACR > 300 mg/g. The factors analyzed using univariate analysis (in preparation for multiple regression) included age, gender, duration of T2DM, BMI, HbA1C, systolic BP, hypertension, and the use of ACEI/ARB. Logistic regression analyses were performed using LVH as the dependent variable to analyze the association between albuminuria and LVH after adjusting for additional variables such as age, gender, albumin, HbA1C, HOMA-IR, systolic BP, hypertension, and the use of ACEI or ARB medication.

For comparison of the variable between/among patients with differing characteristics, Student's *t*-test or Wilcoxon rank-sum test was used for continuous variable, and Chi-square test was used for categorical variable. Numerical variables are presented as means ± standard deviation upon normal distribution and median (interquartile range) otherwise. Categorical variables are presented as percentage. All statistical analyses were performed using SPSS for Windows version 16.0. Statistical significance was defined as *P* < 0.05.

## 3. Results

### 3.1. Sample Characteristics

A total of 333 subjects were included (Tables 1 and 2). The age was 67.5 ± 13.3 years. Disease (T2DM) duration was 7.0 (2.0–12.0) years. Hypertension was present in 232 (69.7%) out of the 333 patients (69.7%). Microalbuminuria and macroalbuminuria were present in 83 and 31 subjects, respectively.

Percentage of the subjects with hypertension and the use of ACEI/ARB were higher in subjects with early DKD (micro- or macroalbuminuria) than those without DKD (no albuminuria). The patients with early DKD also had higher level of serum insulin, HOMA-IR, HbA1c, and ACR and lower level of serum albumin. Patients with early DKD seemed to have longer disease duration, but the difference was not statistically different.

With the subjects with early DKD, those with macroalbuminuria had higher HbA1c and lower serum albumin than those with microalbuminuria. Subjects with microalbuminuria did not differ from those with macroalbuminuria in age, disease duration, systolic BP, BMI, HOMA-IR, creatinine, blood lipid profiles, and the use of ACEI/ARB.

### 3.2. LVMI and LVH

Upon the initial step (univariate analysis) of the stepwise multiple linear regression (Table 3), higher LVMI was associated with albuminuria status (no albuminuria and microalbuminuria, versus macroalbuminuria), older age, higher systolic BP, and nonuse of ACEI/ARB. A multiple linear regression analysis identified albuminuria status as an independent factor that could influence LVMI, with a regression coefficient at 13.61 (*P* < 0.0001).

In logistic regression analysis, the ORs for left ventricular hypertrophy were higher with increasing albuminuria as presented in Table 4. In the macroalbuminuria subgroup, the OR was 3.940 (95% confidence interval (CI) 1.553–9.993) for left ventricular hypertrophy and the OR in the microalbuminuria subgroup was 2.473 (95% CI 1.370–4.464) after adjusting for age, gender, albumin, HbA1c, hypertension, and the use of ACEI or ARB medication.

Apparently, greater LVMI corresponded to higher severity of cardiac hypertrophy. In patients with non-DKD, LVMI was 136.7 ± 17.6 g/m^2^ in patients with LVH and 96.3 ± 14.6 g/m^2^ in those without LVH (*P* < 0.01). In patients with early DKD, LVMI was 148.7 ± 26.1 g/m^2^ in patients with LVH and 99.9 ± 13.3 g/m^2^ in those without LVH (*P* < 0.01).

### 3.3. LV Geometry

Table 5 summarises echocardiographic measurements by the presence of DKD. LVSD, PWTD, RWT, and LVMI of early DKD were significantly increase than those of non-DKD, but the important indicators of systolic function (ejection fraction(EF) and fraction shortening(FS)) were not significant difference between non-DKD and early DKD. LVIDS, LVSD, PWTD, and RWT were higher and FS were lower in macroalbuminuria subgroup, but these were not different between non-DKD and microalbuminuria groups.

Among patients with early DKD, LVMI were higher in macroalbuminuria, compared with microalbuminuria group (137.0 ± 35.6 versus 124.3 ± 30.6), but it did not reach statistical difference (*P* = 0.062). LVIDD in macroalbuminuria group, not LVSD, PWTD, and RWT, increased evidently. Surprisingly, our study showed that FS were lower in macroalbuminuria compared with microalbuminuria subgroup.

Abnormal pattern of LV geometry was concentric hypertrophy (36.0%), followed by eccentric hypertrophy (21.0%) in subjects with early DKD (Table 5). The percentage of eccentric hypertrophy seemed to be higher in subjects with macroalbuminuria (29.0%) versus those with microalbuminuria (18.1%).

## 4. Discussion

In the current study, we found a clear association between increased LVMI (as well as the rate of LVH) and albuminuria status (no albuminuria and microalbuminuria versus macroalbuminuria) in T2DM patient with minimal loss of renal function. Such an association is independent of other variates that influence LVMI. As a matter of fact, the association of both LVMI and LVH with albuminuria status had much higher coefficient than any other risk factors (e.g., age, hypertension, and the use of ACI/ARB).

Over the past few years, the association between albuminuria and LVH has been reported. Nguyen et al. reported an independent association of LVH with microalbuminuria (OR 2.0 [1.4–2.9]) and even higher level of association in older patients with both CAD and microalbuminuria [16]. Liu et al. showed a higher rate of LVH (49%) in T2DM patients with macroalbuminuria than that (31%) in patients with microalbuminuria and in those with no albuminuria (23%) [17]. A prospective cohort study from Germany pointed out that a significant association of urinary ACR at baseline with increase in LVM over the following 5 years in a population-based sample of individuals aged 45 years and older, which also emphasized urinary ACR, was independent of other common cardiovascular risk factors and applicable to both genders [18]. However, most previous studies focused on patients with predisposing factors for cardiovascular disease such as hypertension, obesity, high thyroid hormone status, and late chronic kidney disease (CKD stages 3–5). Our study chooses a highly selected population (DKD patients without decreasing GFR) which is extremely easy to overlook in our clinical work. The findings suggest that albuminuria status could be used, even in the early state of DKD, as a marker for subclinical damage and, particularly, the risk for cardiovascular events. Our findings provided a link between the prognostic value of urinary ACR and LVH in early DKD.

Accumulation of advanced glycation end products (AGEs), activation of protein kinase C (PKC), transforming growth factor-*β*1 (TGF-*β*1), reactive oxygen species (ROS) which are closely related to the development of DKD also involved into the occurrence and progression of left ventricular hypertrophy [19]. Activation of these pathways mentioned above affect myocardial energy metabolism, decease activity of ROS metabolic-related enzyme and cause cardiovascular dysfunction, cardiomyocyte hypertrophy, and excessive accumulation of collagen fibers in the myocardium [20]. It can be said that renal damage, persistent albuminuria, and LVH share some similar mechanisms. Our results proved that the association between increased LVMI and albuminuria status existed not only in CKD stages 3–5 patients but also in early DKD patients (CKD stages 1-2). The high activity of renin-angiotensin-aldosterone system (RAAS) in early DKD may be an important reason why the relationship between LVH and proteinuria began to unravel even in patients with early renal injury [21]. Nowadays, involvement of both kidneys and the cardiovascular system is common in conjunction with T2DM. The association between cardiac and renal disease has been well described and these complex interactions have been captured with the emergence of the concept of cardiorenal syndrome (CRS) [21, 22].

LVH is a pathological change that precedes/underlies ischemic heart disease, arrhythmias, and congestive heart failure [23]. In the current study, the rate of LVH was 32.9% in T2DM patients without early DKD. The result was consistent with the HyperGEN Study [24], which showed that the rate of LVH was 38.0% and diabetic patients had higher LVM compared with nondiabetic patients. Currently, scant information is available in the literature about the influence of diabetes on LV structure and function in patients with renal damage. A study by Nardi et al. [25] focused on this issue in a sample of 288 patients with CKD and hypertension (112 of whom had diabetes). In this study, the rate of LVH in diabetic patients with CKD was as high as 63.39% and diabetic patients had a very high prevalence of concentric LVH (52/112, 46.4%). The rate of LVH in our study was lower than previous study, because we only enroll patients with >60 mL min/1.73 m^2^, but our study also showed the high rates of LVH and concentric LVH in patients with renal damage. Concentric LVH is associated with impairment of diastolic function, which has been demonstrated to increase CVD risk independently from left ventricular mass and BP levels [26]. These data indicate that the majority of patients with early DKD already have high CVD risk.

The correlation between blood pressure and LVH in patients with T2DM and CKD has been demonstrated in previous studies [27, 28]. Our study confirmed that LVH is more frequent in hypertensive patients (46.6% versus 28.7%; *P* = 0.002). Through multivariable analysis, we failed to show a statistically significant association of systolic blood pressure (SBP) with LVMI (*P* = 0.0578) but believe that such an association could be revealed with studies of larger sample size. Our study also confirmed an association of lower LVMI with the use of ACEI/ARB (112.1 ± 29.1 versus 120.4 ± 27.8 g/m^2^; *P* = 0.008). Nowadays, use of pharmacologic strategies with interruption of the RAAS with ACE inhibitors or ARBs is a primary risk-reduction strategy [21]. Our results were consistent with previous studies, but we did not analyze the function of certain drug among ACEI/ARB because of insufficient number of subjects. Age is another important factor in the relationship between albuminuria and LVH. To elderly populations (age > 65), the rate of LVH peaked to 47.5% (*P* = 0.005). Multivariable analysis revealed age was related with LVMI (*P* = 0.0005), which was consistent with previous clinical trial [29]. As for factors that increase the preload, such as obesity and renal dysfunction, they have also been associated with LVH [30]. However, we failed to find an association of either LVMI or LVH with BMI. The reason for this result might be that most of patients included were with lower BMI than westerners generally. 86.7% of patients had a BMI lower than 28 kg/m^2^. Secondly, although metabolic syndrome (MetS) maintains its role as a risk factor for LVH independently of age and systolic BP in clinical practice [31], the effects of MetS on LVH are mainly driven by the degree of abdominal adiposity. BMI is not an accurate assessment of abdominal obesity.

The current study was conducted in a sample of modest size. Accordingly, the results need validation by future studies. The factors that influenced LVMI versus LVH were slightly different: albuminuria, hypertension, and the use of ACEL/ARB were factors that influenced both LVMI and LVH; however, LVMI was influenced by age and not gender, but LVH was influenced by gender and not age. The albuminuria status is imbalanced: there were much more subjects with no albuminuria. This, however, may represent the real-world situation. In addition, the current study is cross-sectional by design and thus could not establish a cause-effect relationship.

## 5. Conclusion

In summary, the results of the current study extended previous identified association of albuminuria with LVH in T2DM patients to a relative early stage of DKD. In addition, we confirmed that higher level of albuminuria (macroalbuminuria versus microalbuminuria) is associated with increasing LVMI and LVH rate. These results encourage the use of albuminuria as a surrogate marker for cardiovascular diseases in T2DM patients.

## Figures and Tables

**Table 1:** The main clinical and biochemical characteristics of type 2 diabetic patients with or without early diabetic nephropathy.

Characteristics	Non-DKD	Early DKD	*P* value
	(*n* = 219)	(*n* = 114)	
Age (year)	66.7 ± 13.9	68.9 ± 12.1	0.141
Male, *n* (%)	138 (63.0)	67 (58.7)	0.477
Hypertension, *n* (%)	144 (65.8)	88 (77.2)	**0.020**
ACEI/ARB use, *n* (%)	88 (40.2)	61 (53.5)	**0.020**
Smoking (%)	19 (8.7)	13 (11.4)	0.440
Duration of diabetes (yr)	7.0 (2.0–11.0)	10.0 (4.0–15.0)	0.105
SBP (mmHg)	132.5 ± 17.4	135.9 ± 16.7	0.085
DBP (mmHg)	78.0 ± 10.5	79.9 ± 11.4	0.141
BMI (kg/m^2^)	24.4 ± 3.6	24.1 ± 3.9	0.493
eGFR (mL/min/1.732)	91.7 (78.5–107.2)	87.8 (73.0–105.3)	0.079
Laboratory			
Glucose (mmol/L)	6.9 (5.6–8.6)	7.0 (5.9–9.4)	0.327
Insulin (*μ*U/mL)	8.7 (5.6–14.9)	11.4 (6.6–16.8)	**0.017**
HOMA-IR	2.5 (1.6–4.7)	3.8 (2.1–6.7)	**0.012**
HbA1c (mean ± SD, %)	7.9 ± 2.0	8.7 ± 2.2	**0.002**
Albumin (g/L)	40.8 ± 4.0	38.4 ± 4.9	**<0.001**
Uric acid (*μ*mol/L)	0.322 ± 0.092	0.325 ± 0.088	0.0798
Creatinine (*μ*mol/L)	72.1 ± 16.1	73.7 ± 16.7	0.391
ACR (mg/g)	8.5 (5.9–14.6)	140.2 (53.7–466.4)	**<0.001**
Triglycerides (mmol/L)	1.5 (1.0–2.2)	1.5 (1.2–2.2)	0.459
Total cholesterol (mmol/L)	4.6 (4.0–5.4)	4.8 (4.2–5.4)	0.641
HDL cholesterol (mmol/L)	1.1 (0.9–1.3)	1.0 (0.9–1.2)	0.086
LDL cholesterol (mmol/L)	2.6 ± 0.8	2.8 ± 0.9	0.083

ACEI/ARB: angiotensin converting enzyme inhibitor/angiotensin receptor blocker; eGFR: modification of diet in renal disease study, glomerular filtration rate; ACR: albumin-to-creatinine ratio; BMI: body mass index; SBP: systolic blood pressure; DBP: diastolic blood pressure.

Data are means SD, median (25–75%), or number (percent).

**Table 2:** The main clinical and biochemical characteristics of type 2 diabetic patients with early diabetic nephropathy.

Characteristics	Microalbuminuria	Macroalbuminuria	*P* value
	(*n* = 83)	(*n* = 31)	
Age (year)	69.4 ± 11.9	67.9 ± 12.7	0.556
Male, *n* (%)	48 (57.8)	19 (61.3)	0.832
Hypertension, *n* (%)	64 (77.1)	24 (77.4)	0.972
ACEI/ARB use, *n* (%)	45 (54.2)	16 (51.6)	0.804
Smoking (%)	6 (7.23)	7 (22.6)	0.022
Duration of diabetes (yr)	9.0 (2.0–14.0)	10.0 (4.0–18.0)	0.270
SBP (mmHg)	134.3 ± 17.4	140.2 ± 14.0	0.096
DBP (mmHg)	79.8 ± 11.4	80.3 ± 11.8	0.841
BMI (kg/m^2^)	23.9 ± 3.9	24.7 ± 4.0	0.383
eGFR (mL/min/1.732)	87.4 (74.7–109.1)	88.4 (69.4–100.8)	0.205
Laboratory			
Glucose (mmol/L)	6.8 (5.9–9.2)	7.2 (6.0–11.9)	0.352
Insulin (*μ*U/mL)	11.5 (6.9–17.5)	11.0 (5.6–18.8)	0.647
HOMA-IR	3.7 (2.0–6.3)	3.8 (2.3–7.3)	0.782
HbA1c (mean ± SD, %)	8.5 ± 2.3	9.2 ± 1.9	**0.046**
Albumin (g/L)	39.3 ± 4.5	36.0 ± 5.3	**<0.001**
Uric acid (*μ*mol/L)	0.326 ± 0.089	0.321 ± 0.088	0.809
Creatinine (*μ*mol/L)	72.4 ± 16.4	77.2 ± 17.1	0.174
ACR (mg/g)	91.6 (45.7–175.1)	927.6 (535.8–1525.5)	**<0.001**
Triglycerides (mmol/L)	1.6 (1.2–2.3)	1.4 (1.0–2.2)	0.462
Total cholesterol (mmol/L)	4.9 (4.2–5.4)	4.5 (4.0–5.5)	0.839
HDL cholesterol (mmol/L)	1.0 (0.9–1.2)	1.0 (0.9–1.3)	0.878
LDL cholesterol (mmol/L)	2.8 ± 0.8	2.8 ± 1.0	0.840

ACEI/ARB: angiotensin converting enzyme inhibitor/angiotensin receptor blocker; eGFR: modification of diet in renal disease study, glomerular filtration rate; ACR: albumin-to-creatinine ratio; BMI: body mass index; SBP: systolic blood pressure; DBP: diastolic blood pressure.

**Table 3:** The stepwise multiple linear regression for LVMI in patients with type 2 diabetes.

Variable	Standardized coefficients	Standard error	*t* value	*P*
Albuminuria status	13.61266	2.19647	6.20	<0.0001
Age (per decade years)	0.38304	0.10872	3.52	0.0005
SBP	0.16585	0.08709	1.90	0.0578
ACEI/ARB use (no versus yes)	−16.57811	3.57836	−4.63	<0.0001

ACEI/ARB: angiotensin converting enzyme inhibitor/angiotensin receptor blocker.

**Table 4:** Adjusted ORs and 95% CIs for LHV according to albuminuria.

		ORs (95% CI)		*P* value
	Non-DKD	Microalbuminuria	Macroalbuminuria	
Model 1	1.0	2.164 (1.275–3.672)	4.505 (1.962–10.343)	<0.001
Model 2	1.0	2.479 (1.381–4.449)	3.828 (1.516–9.665)	<0.001
Model 3	1.0	2.473 (1.370–4.464)	3.940 (1.553–9.993)	<0.001

Model 1 adjusted for age and gender.

Model 2 further adjusted for HbA1c and albumin.

Model 3 further adjusted for hypertension and the use of ACEI or ARB medication.

**Table 5:** The echocardiographic data and LV geometry of type 2 diabetic patients in our study.

Characteristics	The presence of early DKD			Early DKD		
	Non-DKD	Early DKD	*P* value	Microalbuminuria	Macroalbuminuria	*P* value
	(*n* = 219)	(*n* = 114)		(*n* = 83)	(*n* = 31)	
LV dimension						
LVIDD (mm)	47.1 ± 4.0	47.7 ± 4.7	0.183	47.2 ± 4.3	49.0 ± 5.1	0.059
LVIDS (mm)	29.7 ± 3.3	30.6 ± 4.2	**0.027**	30.0 ± 3.5	32.3 ± 5.4	**0.009**
IVSD (mm)	10.3 ± 1.5	11.2 ± 1.6	**<0.001**	11.1 ± 1.6	11.3 ± 1.5	0.565
PWTD (mm)	9.5 ± 1.2	10.2 ± 1.5	**<0.001**	10.1 ± 1.4	10.4 ± 1.5	0.241
RWT	0.41 ± 0.06	0.43 ± 0.08	**0.004**	0.43 ± 0.08	0.43 ± 0.07	0.924
LVMI (g/m^2^)	109.6 ± 24.6	127.7 ± 32.40	**<0.001**	124.3 ± 30.6	137.0 ± 35.6	0.062
EF (%)	65.7 ± 6.9	64.7 ± 6.9	0.205	65.4 ± 6.5	62.6 ± 7.3	**0.052**
FS (%)	37.0 ± 4.6	35.9 ± 4.85	0.057	36.5 ± 4.3	34.4 ± 6.0	**0.039**
LV geometry						
Normal (%)	103 (47.0)	34 (29.8)		28 (33.7)	6 (19.4)	
Concentric remodeling (%)	44 (20.0)	15 (13.2)		11 (13.3)	4 (12.9)	
Concentric hypertrophy (%)	33 (15.2)	41 (36.0)		29 (34.9)	12 (38.7)	
Eccentric hypertrophy (%)	39 (17.8)	24 (21.0)		15 (18.1)	9 (29.0)	
LVH, *n* (%)	72 (32.9)	65 (57.0)	**<0.001**	44 (53.0)	21 (67.7)	0.157

LV: left ventricle; LVMI: left ventricle mass index; LVIDD: left ventricular internal diameter, diastolic; LVIDS: left ventricular internal diameter, systolic; IVSD: interventricular septum, diastolic; PWTD: posterior wall thickness, diastolic; RWT: relative wall thickness; LVMI: left ventricular mass index; EF: ejection fraction; FS: fraction shortening.

Data are means SD, median (25–75%), or number (percent).
